# Which of the nine facial profiles according to AM Schwarz is the most (un)attractive?

**DOI:** 10.1007/s00056-025-00584-z

**Published:** 2025-04-08

**Authors:** F. Kunz, N. F. Nordblom, B. Goetz, L. Fenger, A. Stellzig-Eisenhauer

**Affiliations:** Department of Orthodontics, University Hospital Würzburg, Pleicherwall 2, 97070 Würzburg, Germany

**Keywords:** Attractiveness, Orthodontics, Diagnostic analysis, Three-dimensional face scans, Basel face model, Attraktivität, Kieferorthopädie, Diagnostische Analyse, Dreidimensionale Scans des Gesichts, Basler Gesichtsmodell

## Abstract

**Objective:**

The human profile has always been fascinating to artists, scientists, and physicians. In the mid 20th century, AM Schwarz created a profile analysis for orthodontics, which categorizes both the sagittal position of the midface as well as the position of the chin, and is still widely used today. In combination, this analysis leads to nine different profile types, which are assumed to differ in terms of their attractiveness. Since there has been hardly any scientific research in this area so far, the aim of this study was to quantify the attractiveness of the nine profile types according to AM Schwarz.

**Methods:**

The “Basel face model” is a three-dimensional (3D) facial dataset that was created artificially by morphing a total of 100 female and 100 male 3D face scans. For the present study, this face model was modified to ideally represent all nine profile types according to AM Schwarz. The representation of those nine facial models depicted in a standardized lateral perspective were assessed in terms of attractiveness by 1261 volunteers of different ages and educational level. The ratings were statistically analyzed using repeated measures analysis of variance (ANOVA) and pairwise comparisons.

**Results:**

The results revealed significant differences in attractiveness regarding the nine profile types. Average faces were perceived significantly more attractive than antefaces and these in turn were perceived more attractive than retrofaces. Furthermore, straight and backward-slanting profiles were rated significantly more attractive than forward-slanting profiles. There were no clinically relevant differences between the assessments of males and females or between raters of different educational levels.

**Conclusion:**

The perceived attractiveness of the nine profile types according to AM Schwarz differed significantly. This perception did not seem to be affected by gender or the level of education.

## Abstract (de)

**Ziel:**

Das menschliche Profil strahlt seit jeher eine Faszination für Künstler, Wissenschaftler und Mediziner aus. Mitte des 20. Jahrhunderts erstellte A.M. Schwarz eine Profilanalyse für die Kieferorthopädie, welche sowohl die sagittale Position des Mittelgesichtes als auch die des Kinns beschreibt und noch heute weit verbreitet Anwendung findet. Kombiniert ergeben sich bei dieser Analyse 9 verschiedene Profiltypen, welche sich mutmaßlich hinsichtlich ihrer Attraktivität unterscheiden. Da es diesbezüglich bislang kaum wissenschaftliche Untersuchungen gibt, war es das Ziel dieser Studie, die Attraktivität der 9 Profiltypen nach A.M. Schwarz zu quantifizieren.

**Material und Methode:**

Das „Basler Gesichtsmodell“ ist ein 3D-Gesichtsdatensatz, welcher durch das Morphen von insgesamt 100 weiblichen und 100 männlichen 3D-Gesichtsscans artifiziell erstellt wurde. Für die vorliegende Studie wurde dieses Gesichtsmodell so angepasst, dass alle 9 Profiltypen nach A.M. Schwarz ideal repräsentiert wurden. Diese 9 Gesichtsmodelle wurden von 1261 Probanden verschiedenen Alters und unterschiedlicher Bildungslevel hinsichtlich der Attraktivität bewertet. Die Einschätzungen wurden mittels ANOVA („analysis of variance“) mit Messwiederholung sowie paarweisen Vergleichen statistisch analysiert.

**Ergebnisse:**

Es zeigten sich signifikante Attraktivitätsunterschiede bezüglich der 9 Profiltypen. Durchschnittsgesichter wurden signifikant attraktiver als Vorgesichter und diese wiederum attraktiver als Rückgesichter wahrgenommen. Weiterhin wurden gerade und nach hinten schiefe Profile signifikant attraktiver bewertet als nach vorne schiefe Profile. Zwischen den Einschätzungen männlicher und weiblicher Probanden sowie zwischen denen durch Probanden mit unterschiedlichem Bildungslevel zeigten sich keine klinisch relevanten Unterschiede.

**Schlussfolgerungen:**

Zwischen den 9 Profiltypen nach A.M. Schwarz bestanden signifikante Attraktivitätsunterschiede. Diese Wahrnehmung schien weder vom Geschlecht noch vom Bildungslevel abhängig zu sein.

## Introduction

The human profile has always been fascinating to artists, scientists, and physicians. Important artists of the Renaissance such as Leonardo da Vinci and Albrecht Dürer intensively focused on the human face when creating their portraits, searching for perfect facial proportions to reveal and quantify the secret of facial beauty. It was about this time (around the 15–16th century) that anthropology was first mentioned and, thus, an increasing number of scientific measurements of the human body and facial proportions were performed [1]. In the 19th century, measurements of the face were then used to gain better knowledge of human variation and to classify human faces.

Even today, measuring faces remains an important aspect of diagnostics and treatment planning in some fields of modern medicine, such as maxillofacial surgery and orthodontics [2, 3]. Especially in the latter, analysis of lateral photographs is a fundamental part of orthodontic standard diagnostics, as orthodontic treatment affects the soft tissue profile of young patients by influencing growth of the jaws as well as by altering the inclination of the incisors [4–12]. Over the last few decades, a variety of different methods for measuring and analyzing facial profiles have been proposed in order to assess orthodontically relevant aspects of the profile [2, 3, 13, 14]. Besides common international methods, which were introduced by well-known orthodontists such as Ricketts, Legan and Burstone as well as Holdaway, the analysis according to AM Schwarz is particularly popular in German-speaking countries [4, 15].

**Fig. 1:**
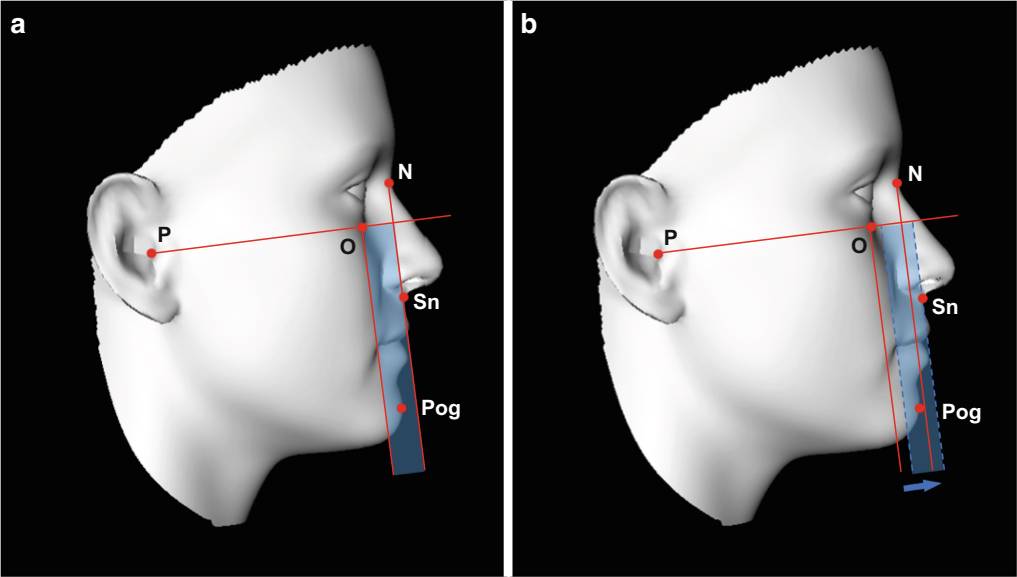
Illustration of the geometric constructions for the profile analysis according to AM Schwarz. The jaw profile field (area below the horizontal line between the two perpendicular lines) is shaded blue. **a** Straight average face; **b** straight anteface with a parallelly shifted jaw profile field Darstellung der geometrischen Konstruktionen für die Profilanalyse nach A.M. Schwarz. Das Kieferprofilfeld (Fläche unterhalb der horizontalen Linie zwischen den beiden Senkrechten) ist blau schattiert. **a** Gerades Durchschnittsgesicht; **b** gerades Vorgesicht mit parallel verschobenem Kieferprofilfeld

For the profile analysis according to AM Schwarz (Fig. 1), the first step is to construct a horizontal line on a well-aligned lateral photo, which is defined by the two landmarks porion (P, highest point of the cutaneous auditory meatus) and orbitale (O, corresponds to the bony orbital ridge and is located one palpebral width below the unforced open eye). In the second step, perpendiculars to this horizontal line are constructed through the landmarks O and nasion (N, most posterior point of the curvature at the base of the nose). According to AM Schwarz, an ideal sagittal position of the midface is given when the landmark subnasale (Sn, transition of the columella to the upper lip) is located exactly on the perpendicular through N. The corresponding profile is then labelled as an “average face”. If Sn and therefore the midface is located anteriorly or respectively posteriorly to this perpendicular, the profile is referred to as an “anteface” or a “retroface”, respectively. To assess the position of the chin, the so-called “jaw profile field” is defined, which describes the area below the horizontal line between the two perpendiculars. In case of an ante- or retroface, both perpendiculars and thus the jaw profile field are parallelly shifted in relation to the horizontal line until Sn is again located on the shifted nasion perpendicular. The ideal sagittal position of the chin is defined according to AM Schwarz when the cutaneous pogonion (Pog, most anterior point of the chin contour) is located exactly in the middle of the (shifted) jaw profile field and the corresponding profile is then referred to as a “straight profile”. In case that the landmark Pog of a given profile is situated more anteriorly or posteriorly, the corresponding profile is labeled as a “forward- or backward-slanting profile”. In combination, this analysis leads to nine profile types and describes both the sagittal position of the midface as well as the position of the chin [16].

AM Schwarz stated that straight antefaces as well as straight retrofaces are esthetically equal to straight average faces, whereas all types of forward or backward-slanting profiles are esthetically less favorable compared to straight profiles [16]. This may lead to the conclusion that orthodontic treatment should always aim to achieve straight profiles by the end of treatment. On the other hand, there are only a few studies so far investigating the impact on attractiveness of the nine profile types according to AM Schwarz [4, 15]. This seems particularly surprising given the fact that the profile analysis according to AM Schwarz is very common in Germany and the analysis of photographs is even part of standard orthodontic diagnostics.

Therefore, the aim of the present study was to investigate whether the nine profiles according to AM Schwarz differ in terms of their perceived attractiveness. Moreover, the extent to which additional factors such as gender and level of education of the raters might affect this perception was examined.

## Methods

This study was designed as an observational study, carried out in compliance with the Declaration of Helsinki and approved by the Ethics Committee of the University Hospital of Würzburg (33/18). All participants, as well as their legal guardians in case of minors provided their written informed consent to participate in the investigation.

### Presentation of the profiles according to AM Schwarz

The “Basel face model” is a three-dimensional (3D) facial dataset that was created artificially by morphing a total of 100 female and 100 male 3D face scans [17]. This face model represented the baseline situation for the presentation material in this study. Using the software scalismo (Graphics and Vision Research group, University of Basel in collaboration with Shapemeans GmbH, Allschwil, Switzerland), this face model was modified to represent all nine profile types according to AM Schwarz.

For this purpose, the upper third of the face was left unaltered, ensuring no modifications were made in this area. Subsequently, the middle and lower thirds of the face were modified to represent all nine profile types according to AM Schwarz in a clinically realistic manner. The first step in this process was to construct the straight average face model, with Sn positioned precisely on the perpendicular through N and Pog placed exactly at the center of the jaw profile field (the area between the perpendicular lines through N and O, as shown in Fig. 1a). Next, the antefaces and retrofaces were created by adjusting the morphology of the face model. These adjustments resulted in a shift of the sagittal position of the midface anteriorly (antefaces) and posteriorly (retrofaces). The extent of these adjustments was in the range of 1/3 the width of the jaw profile field. The jaw profile field was then shifted to the altered position of the midface (blue area in Fig. 1b) until Sn again was located on the shifted perpendicular through N.

**Fig. 2:**
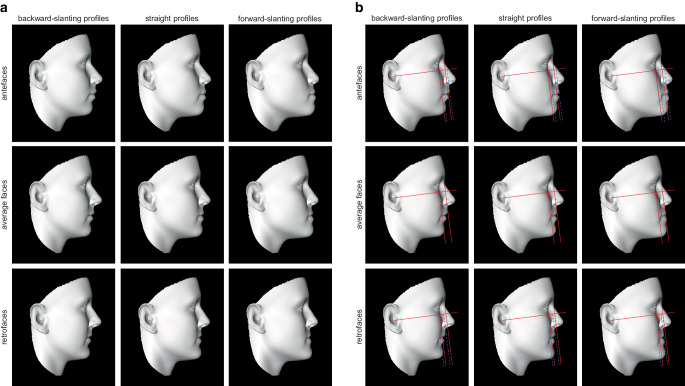
Illustration of the nine profile types according to AM Schwarz without (**a**) and with (**b**) geometric constructions (red: horizontal line defined by the landmarks porion and orbitale, as well as the perpendiculars through the orbitale and nasion; blue: parallel shifted perpendiculars in case of ante- and retrofaces) Darstellung der 9 Profiltypen nach A.M. Schwarz ohne (**a**) und mit (**b**) geometrischen Konstruktionen (rot: horizontale Linie, die durch die Landmarken Porion und Orbitale definiert ist, sowie die Senkrechten durch Orbitale und Nasion; blau: parallel verschobene Senkrechten bei Vor- und Rückgesichtern)

In addition to these changes, the lower third of the face model was adjusted for each profile (anteface, average face, and retroface) to correspond to the three different sagittal positions of the chin, as outlined by AM Schwarz. As a result, Pog was always positioned at the center of the (shifted) jaw profile field for all straight profiles. For backward-slanting faces Pog nearly touched the (shifted) perpendicular though O, while for forward-slanting profiles, it was close to the (shifted) perpendicular through N. All nine resulting profiles are depicted in Fig. 2.

### Preparation of an evaluation sheet and data collection

To record assessments of attractiveness, evaluation sheets were created on which all nine profile types were presented next to each other in random order. All test subjects, recruited on a voluntary basis at the University Hospital of Würzburg, were asked to rank these nine profiles according to their subjectively perceived attractiveness. A rating of 1 corresponds to the most attractive profile and a rating of 9 to the least attractive profile. Age, gender, and educational level were recorded on the same evaluation sheet. This sheet was given to the test subjects as a printed copy in DIN A3 size (29.7 cm × 42 cm) and the test subjects were instructed to complete it independently which took about 3–5 min. After being returned, the evaluation forms were digitized and prepared for statistical analysis.

### Statistical analysis

Statistical analysis of the data was carried out by a professional statistician (Statworx, Frankfurt am Main, Germany) using IBM® SPSS® Statistics Version 25 for Windows (IBM, Armonk, NY, USA).

Descriptive data analysis was performed by specifying the sample size (*N*), mean values (M) as well as standard deviations (SD). The evaluations for the three face types and the three chin positions were aggregated by averaging the three possible configurations for each category. The comparisons of the assessments of the (aggregated) profile types were performed by means of repeated measures analysis of variance (ANOVA) with correction according to Greenhouse–Geisser. To this end, we assumed the ratings of the profiles to be quasi-metric. Subsequently, pairwise comparisons including Bonferroni correction in terms of a post hoc analysis were carried out for detailed differentiation.

To examine the impact of the rater’s gender on the results, *t*-tests for independent samples were calculated indicating the mean difference, 95% confidence interval (CI) and *p*-value (*p*). On the other hand, the impact of the rater’s educational level on the results was examined using mixed ANOVA with correction according to Greenhouse–Geisser and again, pairwise comparisons including Bonferroni correction were carried out.

The level of significance was set at 5% for all statistical analyses.

## Results

### Comparison of the ratings of the nine profile types

**Fig. 3:**
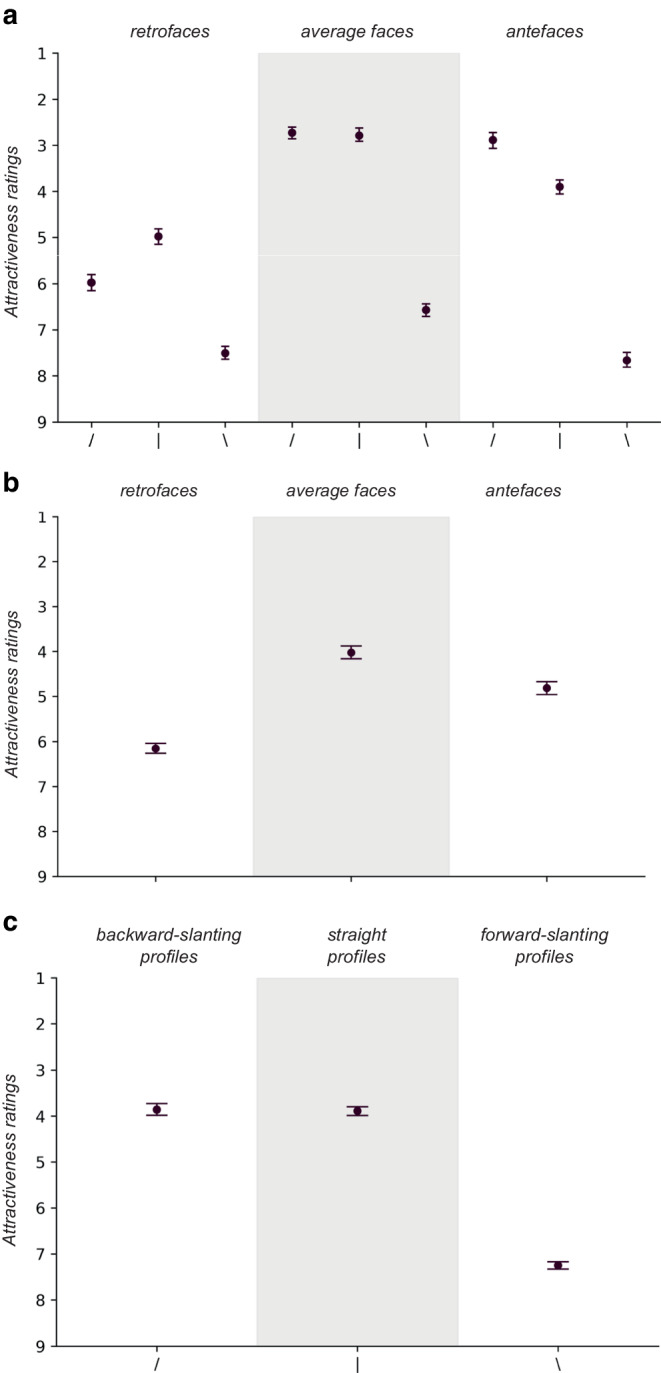
**a** Attractiveness ratings for the nine profile types according to AM Schwarz. Error bars indicate the mean ratings as well as the upper and lower limits of the 95% confidence intervals (CI). / = backward-slanting profile, | = straight profile and \ = forward-slanting profile. **b** Combined attractiveness ratings for the three face types (retrofaces, average faces, and antefaces). Error bars indicate the mean ratings as well as the upper and lower limits of the 95%CI.** c** Combined attractiveness ratings for the three positions of the chin (backward-slanting, straight, and forward-slanting profiles). Error bars indicate the mean ratings as well as the upper and lower limits of the 95%CI **a** Attraktivitätsbewertungen für die 9 Profiltypen nach A.M. Schwarz. Die Fehlerbalken zeigen den Mittelwert der Bewertungen sowie die obere und untere Grenze des 95%-Konfidenzintervalls an. / = nach hinten schiefes Profil, | = gerades Profil und \ = nach vorne schiefes Profil. **b** Zusammengefasste Attraktivitätsbewertungen für die 3 Gesichtstypen (Rückgesichter, Durchschnittsgesichter und Vorgesichter). Die Fehlerbalken zeigen den Mittelwert der Bewertungen sowie die obere und untere Grenze des 95%-Konfidenzintervalls an. **c** Zusammengefasste Attraktivitätsbewertungen für die 3 Positionen des Kinns (nach hinten schiefe, gerade und nach vorne schiefe Profile). Die Fehlerbalken zeigen den Mittelwert der Bewertungen sowie die obere und untere Grenze des 95%-Konfidenzintervalls an

A total of 1261 (60.9% female/39.1% male) Caucasian test subjects with an average age of 32.2 ± 17.4 years participated in the study. At the time of data acquisition, the highest educational levels achieved by the subjects were as follows: 4.4% had completed primary school, 8.6% had finished lower secondary school, 22.8% had completed middle school, 8.8% had attended vocational school, and 55.4% had graduated from high school. The attractiveness ratings for each of the nine profile types according to AM Schwarz, as well as of the aggregated ratings for face type and chin position are depicted in Table 1 and Fig. 3.

**Table 1:** Descriptive analysis of the attractiveness ratings for the nine profile types according to AM Schwarz, as well as the aggregated ratings for the sagittal position of the midface and chin Deskriptive Analyse der Attraktivitätsbewertungen für die 9 Profiltypen nach A.M. Schwarz sowie der aggregierten Bewertungen für die sagittale Position von Mittelgesicht und Kinn

Profile type	Backward-slanting	Straight	Forward-slanting	Aggregated ratings for the sagittal position of the midface
Anteface	2.88 ± 1.88	3.90 ± 1.88	7.67 ± 1.70	4.82 ± 1.06
Average face	2.73 ± 1.69	2.79 ± 1.51	6.57 ± 1.56	4.03 ± 0.79
Retroface	5.97 ± 2.05	4.98 ± 1.76	7.51 ± 1.63	6.16 ± 1.09
Aggregated ratings for the sagittal position of the chin	3.86 ± 1.12	3.89 ± 0.84	7.25 ± 1.03	

A rating of “1” corresponds to the most attractive rating and a value of “9” to the most unattractive rating

Mean (M) ± standard deviation (SD). Sample size *N* = 1261

The repeated measures ANOVA with Greenhouse–Geisser correction determined statistically significant differences between the ratings of the nine profile types (*F *(6.28, 7917.67) = 1499.79, *p* < 0.001, partial *η*^*2*^ = 0.543). The detailed results of pairwise comparisons of the ratings are depicted in Table 2. With just a few exceptions, all pairwise comparisons were highly significant (*p* < 0.001). No statistically significant differences were found between the ratings of straight average faces with both backward-slanting antefaces (*p* = 1.000) and backward-slanting average faces (*p* = 1.000), as well as between backward-slanting antefaces and backward-slanting average faces (*p* = 0.777). Moreover, no statistically significant differences were found between the ratings of forward-slanting antefaces and forward-slanting retrofaces (*p* =0.570). Straight average faces (M ± SD = 2.79 ± 1.51), backward-slanting average faces (2.73 ± 1.69), and backward-slanting antefaces (2.88 ± 1.88) were rated the most attractive, while forward-slanting antefaces (7.67 ± 1.70) and forward-slanting retrofaces (7.51 ± 1.63) were rated as the most unattractive.

**Table 2:** Comparison of the attractiveness ratings of the nine profile types according to AM Schwarz. Pairwise comparisons with Bonferroni correction as post hoc analysis of the repeated measures analysis of variance (ANOVA) Vergleich der Attraktivitätsbewertungen der 9 Profiltypen nach A.M. Schwarz. Paarweise Vergleiche mit Bonferroni-Korrektur als Post-hoc-Analyse der Varianzanalyse mit wiederholten Messungen (ANOVA)

		Anteface			Average face			Retroface		
		Forward-slanting (7.67 ± 1.70)	Straight (3.90 ± 1.88)	Backward-slanting (2.88 ± 1.88)	Forward-slanting (6.57 ± 1.56)	Straight (2.79 ± 1.51)	Backward-slanting (2.73 ± 1.69)	Forward-slanting (7.51 ± 1.63)	Straight (5.97 ± 2.05)	Backward-slanting (5.98 ± 1.67)
Anteface	Forward-slanting (7.67 ± 1.70)	–	0.001**	0.001**	0.001**	0.001**	0.001**	0.570	0.001**	0.001**
	Straight (3.90 ± 1.88)	0.001**	–	0.001**	0.001**	0.001**	0.001**	0.001**	0.001**	0.001**
	Backward-slanting (2.88 ± 1.88)	0.001**	0.001**	–	0.001**	1.000	0.777	0.001**	0.001**	0.001**
Average face	Forward-slanting (6.57 ± 1.56)	0.001**	0.001**	0.001**	–	0.001**	0.001**	0.001**	0.001**	0.001**
	Straight (2.79 ± 1.51)	0.001**	0.001**	1.000	0.001**	–	1.000	0.001**	0.001**	0.001**
	Backward-slanting (2.73 ± 1.69)	0.001**	0.001**	0.777	0.001**	1.000	–	0.001**	0.001**	0.001**
Retroface	Forward-slanting (7.51 ± 1.63)	0.570	0.001**	0.001**	0.001**	0.001**	0.001**	–	0.001**	0.001**
	Straight (5.97 ± 2.05)	0.001**	0.001**	0.001**	0.001**	0.001**	0.001**	0.001**	–	0.001**
	Backward-slanting (5.98 ± 1.67)	0.001**	0.001**	0.001**	0.001**	0.001**	0.001**	0.001**	0.001**	–

A rating of “1” corresponds to the most attractive rating and a value of “9” to the most unattractive rating

Mean values and standard deviation of the ratings (M ± SD) as well as *p*-values (*p*)

*Significance for *p* < 0.05

**Significance for *p* < 0.01

In terms of aggregated ratings (Fig. 3b, c), our results reveal significant results for both the face type (*F *(1.76, 2224.27) = 1011.74, *p* < 0.001, partial *η*^*2*^ = 0.443) and the position of the chin (*F *(1.81, 2280.73) = 3176.34, *p* < 0.001, partial *η*^*2*^ = 0.716). Average faces (4.03 ± 0.79) were perceived significantly more attractive than antefaces (4.82 ± 1.06/*p* < 0.001) and these in turn significantly more attractive than retrofaces (6.16 ± 1.09/*p* < 0.001). In contrast, straight profiles (3.89 ± 0.84) and backward-slanting profiles (3.86 ± 1.12) were assessed almost equally in terms of perceived attractiveness (*p* = 1.000), with both profiles being perceived significantly more attractive than forward-slanting profiles (7.25 ± 1.03/*p* < 0.001).

### Investigation of contributing factors

**Fig. 4:**
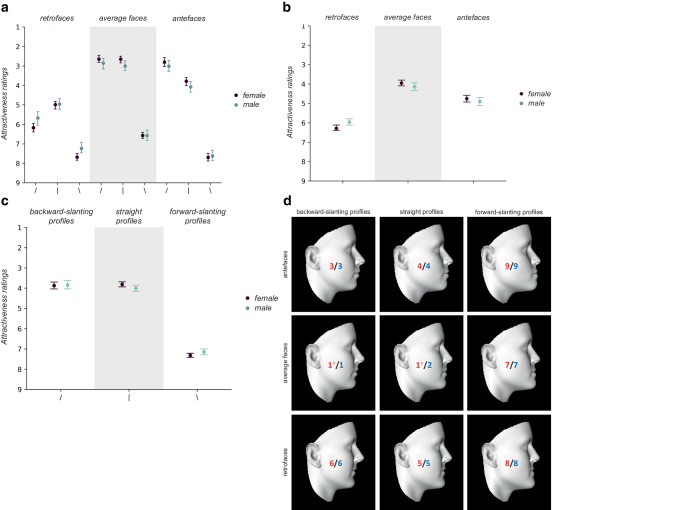
**a** Attractiveness ratings for the nine profile types according to AM Schwarz, stratified according the gender of the evaluators. Error bars show the mean value of the ratings as well as the upper and lower limits of the 95% confidence interval (CI). / = backward-slanting profile, | = straight profile, and \ = forward-slanting profile. **b** Aggregated attractiveness ratings for the three face types (retrofaces, average faces, and antefaces), stratified according to the gender of the evaluators. Error bars show the mean value of the ratings as well as the upper and lower limits of the 95%CI. **c** Aggregated attractiveness ratings for the three positions of the chin (backward-slanting, straight, and forward-slanting profiles), stratified according to the gender of the evaluators. Error bars show the mean value of the ratings as well as the upper and lower limits of the 95%CI. **d** Ranking of the mean attractiveness ratings of women (red number) and men (blue number) for the nine profile types according to AM Schwarz. A rating of ‘1’ corresponds to the most attractive profile and a rating of ‘9’ to the least attractive profile. Error bars show the mean value of the ratings as well as the upper and lower limits of the 95%CI. + The mean values of these two ratings are equal up to the second decimal place, so that the first rank was given twice **a** Attraktivitätsbewertungen für die 9 Profiltypen nach A.M. Schwarz, stratifiziert nach dem Geschlecht der Bewerter. Die Fehlerbalken zeigen den Mittelwert der Bewertungen sowie die obere und untere Grenze des 95%-Konfidenzintervalls an. / = nach hinten schiefes Profil, | = gerades Profil und \ = nach vorne schiefes Profil. **b** Zusammengefasste Attraktivitätsbewertungen für die 3 Gesichtstypen (Rückgesichter, Durchschnittsgesichter und Vorgesichter), stratifiziert nach dem Geschlecht der Bewerter. Die Fehlerbalken zeigen den Mittelwert der Bewertungen sowie die obere und untere Grenze des 95%-Konfidenzintervalls an. **c** Zusammengefasste Attraktivitätsbewertungen für die 3 Positionen des Kinns (nach hinten schiefe, gerade und nach vorne schiefe Profile), stratifiziert nach dem Geschlecht der Bewerter. Die Fehlerbalken zeigen den Mittelwert der Bewertungen sowie die obere und untere Grenze des 95%-Konfidenzintervalls an. **d** Rangfolge der mittleren Attraktivitätsbewertungen von Frauen (rote Ziffer) und Männern (blaue Ziffer) für die 9 Profiltypen nach A.M. Schwarz. Eine Bewertung von „1“ entspricht dem attraktivsten Profil, eine Bewertung von „9“ dem unattraktivsten Profil. + Die Mittelwerte dieser beiden Bewertungen sind bis zur zweiten Dezimalstelle gleich, sodass der erste Rang 2‑mal vergeben wurde

For five of the nine profile types, significant differences were found between the attractiveness ratings given by females and males (Table 3; Fig. 4a). However, the mean differences between the ratings given by both genders were very small throughout. The greatest discrepancy was found for backward-slanting retrofaces with an average deviation of just 0.5 rating points, so that it can be assumed that although these differences were statistically significant, they might be of little clinical relevance. Furthermore, when ranking the mean ratings given by females and males into ascending order, the slightly varying ratings given by both genders did not result in any differences. Comparable results were also found for the aggregated ratings (Table 4, Fig. 4b, c, and 4d).

**Table 3:** Descriptive analysis of the attractiveness ratings for the nine profile types according to AM Schwarz, distinguished according to the gender of the raters Deskriptive Analyse der Attraktivitätsbewertungen für die 9 Profiltypen nach A.M. Schwarz, differenziert nach dem Geschlecht der Bewerter

Profile type		Descriptive results					*t*-test			
		Gender	*N*	M	SD	Ranking	Mean difference	95% CI lower limit	95% CI upper limit	*p*-value
Anteface	Forward-slanting	Female	768	7.69	1.60	9	−0.070	−0.262	0.122	0.475
		Male	493	7.62	1.84	9				
	Straight	Female	768	3.79	1.83	4	0.289	0.077	0.501	0.008**
		Male	493	4.08	1.94	4				
	Backward-slanting	Female	768	2.80	1.79	3	0.211	−0.001	0.424	0.052
		Male	493	3.01	2.01	3				
Average face	Forward-slanting	Female	768	6.57	1.43	7	0.001	−0.175	0.177	0.991
		Male	493	6.57	1.74	7				
	Straight	Female	768	2.65	1.39	1^+^	0.352	0.182	0.523	< 0.001**
		Male	493	3.00	1.67	2				
	Backward-slanting	Female	768	2.65	1.60	1^+^	0.204	0.013	0.395	0.036*
		Male	493	2.85	1.81	1				
Retroface	Forward-slanting	Female	768	7.68	1.54	8	−0.442	−0.625	−0.259	< 0.001**
		Male	493	7.24	1.73	8				
	Straight	Female	768	4.99	1.65	5	−0.032	−0.231	0.167	0.753
		Male	493	4.96	1.92	5				
	Backward-slanting	Female	768	6.17	1.90	6	−0.501	−0.731	−0.271	< 0.001**
		Male	493	5.67	2.22	6				

A rating of “1” corresponds to the most attractive rating and a value of “9” to the most unattractive rating

Descriptive results including gender, sample size (*N*), mean (M), standard deviation (SD) and ranking for the ratings of both genders. Statistical analysis using *t*-tests for independent samples with mean difference, 95% confidence interval (CI) and *p*-value (*p*)

*Significance for *p* < 0.05

**Significance for *p* < 0.01

^+^ The mean values of these two ratings are equal up to the second decimal so that the first rank was given twice

**Table 4:** Descriptive analysis of the attractiveness ratings for sagittal position of the midface and the chin, distinguished according to the gender of the raters Deskriptive Analyse der Attraktivitätsbewertungen für die sagittale Position des Mittelgesichts und des Kinns, differenziert nach dem Geschlecht der Bewerter

Profile type	Descriptive results				*t*-test			
	Gender	*N*	M	SD	Mean difference	95% CI lower limit	95% CI upper limit	*p*-value
Anteface	Female	768	4.76	1.01	0.143	0.023	0.263	0.019*
	Male	493	4.90	1.14				
Average face	Female	768	3.95	0.72	0.186	0.097	0.274	<0.001**
	Male	493	4.14	0.87				
Retroface	Female	768	6.28	1.04	−0.325	−0.447	−0.204	<0.001**
	Male	493	5.96	1.12				
Forward-slanting	Female	768	7.32	0.93	−0.170	−0.286	−0.055	0.004**
	Male	493	7.14	1.15				
Straight	Female	768	3.81	0.79	0.203	0.109	0.297	<0.001**
	Male	493	4.01	0.89				
Backward-slanting	Female	768	3.87	1.10	−0.029	−0.156	0.099	0.660
	Male	493	3.84	1.17				

A rating of “1” corresponds to the most attractive rating and a value of “9” to the most unattractive rating

Descriptive results including gender, sample size (*N*), mean (M) and standard deviation (SD). Statistical analysis using *t*-tests for independent samples with mean difference, 95% confidence interval (CI) and *p*-value (*p*)

*Significance for *p* < 0.05

**Significance for *p* < 0.01

**Fig. 5:**
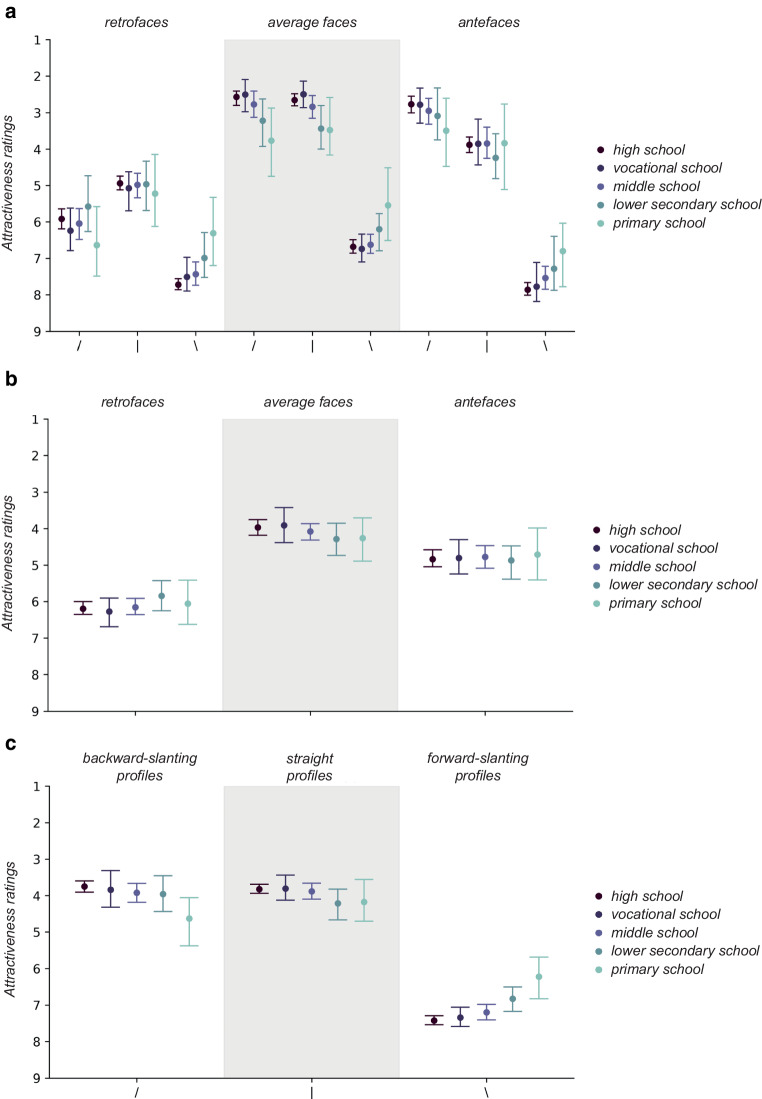
**a** Attractiveness rating for the nine profile types according to AM Schwarz, stratified according to the evaluators’ level of education. Error bars show the mean of the ratings and the upper and lower limits of the 95% confidence interval (CI). / = backward-slanting profile, | = straight profile, and \ = forward-slanting profile. **b** Combined attractiveness ratings for the three face types (retrofaces, average faces, and antefaces), stratified according to the evaluators’ education level. Error bars show the mean of the ratings and the upper and lower limits of the 95%CI. **c** Combined attractiveness ratings for the three positions of the chin (backward-slanting, straight, and forward-slanting profiles), stratified according to the evaluators’ education level. Error bars show the mean of the ratings and the upper and lower limits of the 95%CI **a** Attraktivitätsbewertungen für die 9 Profiltypen nach A.M. Schwarz, stratifiziert nach dem Bildungslevel der Bewerter. Die Fehlerbalken zeigen den Mittelwert der Bewertungen sowie die obere und untere Grenze des 95%-Konfidenzintervalls an. / = nach hinten schiefes Profil, | = gerades Profil und \ = nach vorne schiefes Profil. **b** Zusammengefasste Attraktivitätsbewertungen für die 3 Gesichtstypen (Rückgesichter, Durchschnittsgesichter und Vorgesichter), stratifiziert nach dem Bildungslevel der Bewerter. Die Fehlerbalken zeigen den Mittelwert der Bewertungen sowie die obere und untere Grenze des 95%-Konfidenzintervalls an. **c** Zusammengefasste Attraktivitätsbewertungen für die drei Positionen des Kinns (nach hinten schiefe, gerade und nach vorne schiefe Profile), stratifiziert nach dem Bildungslevel der Bewerter. Die Fehlerbalken zeigen den Mittelwert der Bewertungen sowie die obere und untere Grenze des 95%-Konfidenzintervalls an

The mixed ANOVA revealed a significant impact of the raters’ educational level on the assessment of attractiveness (*F *(25.16, 7705.17) = 5.80, *p* < 0.001, partial η^2^ = 0.019) albeit with rather low effect size. Due to the large number of individual comparisons, the pairwise comparisons of the post hoc analysis are depicted in Fig. 5a. As in the analysis of the influence of the raters’ gender, only slight mean differences were found between the different educational levels so that it can be assumed that the educational level had no clinically relevant impact on the assessments of attractiveness as well. These findings were again applicable to the aggregated ratings (Fig. 5b, c). However, the results revealed that profile types that were generally rated as particularly attractive or unattractive (e.g., straight average faces; backward-slanting average faces and backward-slanting antefaces; or forward-slanting retrofaces and forward-slanting antefaces, respectively) were rated more distinctly by individuals with a higher level of education (vocational school, high school). In contrast, individuals with lower level of education tended to provide more average ratings.

## Discussion

In addition to the analysis of dental models and radiological assessments using cephalometric imaging and orthopantomograms, standard orthodontic diagnostics also includes the analysis of photographs [2, 3]. A crucial component of this diagnostic process is the evaluation of facial esthetics, as enhancing facial esthetics, along with aligning the dental arches and adjusting occlusion, is a fundamental objective of orthodontic therapy [13, 18]. A complete orthodontic photographic analysis requires evaluating photographs taken from various perspectives, typically including a frontal view, a frontal view with a relaxed smile, and a profile view. These images must all be taken in a standardized manner to ensure consistency and accuracy in the assessment. While both frontal views together are used in particular to assess facial symmetry, lip closure, matching of the dental midlines as well as exposure of the teeth when smiling, the main intention of the lateral view is to examine the patient’s profile by analyzing vertical relations of the viscerocranium as well as the sagittal position of the midface and chin [19–21].

In recent decades, various approaches for orthodontically measuring and analyzing facial profiles have been proposed, of which the method according to AM Schwarz is widely used particularly in German-speaking countries [2–4, 13–15, 19]. Based on a geometric construction, this profile analysis distinguishes a total of nine profile types as a result of the combination of three variants of sagittal positions of the midface (referred to as average faces, antefaces, and retrofaces, respectively) as well as three variants of the chin (referred to as straight, forward- and backward-slanting profiles, respectively). AM Schwarz himself gave clear statements regarding the attractiveness of these nine profile types [16]. However, there is limited scientific evidence on the impact of the nine facial types according to AM Schwarz on attractiveness. Therefore, this study was designed to investigate this specific aspect.

There are various research strategies for investigating the perception of attractiveness of facial profiles. The most used methods are the presentation of different profiles by means of silhouettes (sometimes even further reduced to just profile lines) or lateral photographs in which the profile had been artificially altered by means of image processing [22]. Each method has distinct advantages and disadvantages that must be considered and discussed. Some authors advocate for the use of lateral photographs, as this approach provides a more realistic and comprehensive representation of the profile. This method captures a range of abstract factors, such as skin tone, texture, and hair, which contribute to the overall perception of profile attractiveness. Consequently, it leads to more reliable and authentic assessments [4, 6, 8, 23–27]. Nevertheless, this strategy may also introduce bias due to the consideration of these extraneous elements. In contrast, silhouettes reduce the lateral view to its fundamental outline, thereby, eliminating confounding variables and facilitating a more focused assessment of specific features, such as the sagittal position of the midface and the chin. Consequently, this method reduces bias but may omit important details necessary for a comprehensive esthetic evaluation [12, 26, 26, 28, 28–35]. A comparative analysis of the two methods revealed that both methods deliver almost identical results [36]. However, significant differences between the two methods were observed by other authors using a similar study design [24]. Therefore, it is likely that both methods emphasize different aspects of esthetics.

The goal of our investigation was to combine the advantages of both methods. While maintaining realistic proportions, distracting features such as hair and skin tones should be excluded. For this purpose, we decided to adopt a new approach for assessing the attractiveness of profiles using the Basel face model which was developed at the University of Basel to create realistic 3D representations of human faces [17]. Originally, the Basel face model was based on 200 real 3D face scans, which were combined using various statistical methods. Due to the possibility to adjust a variety of different parameters and landmarks, it is possible to synthesize almost any kind of face based on this dataset. In our study, we created all nine profile types according to AM Schwarz by selectively adjusting the midface and chin areas, while leaving the upper third of the face unchanged. This approach allowed us to focus on the relevant regions without affecting other areas, ensuring accuracy of our data [37, 38]. Our approach therefore combines both the advantages of reducing bias that can be achieved by representing profile silhouettes, while preserving the human profile in a much more realistic and natural manner. To our knowledge, this study is the first to quantify the attractiveness of facial profiles using this three-dimensional Basel facial model.

In his textbook, AM Schwarz assumed that (1) all three variants of straight profiles are basically equivalent in terms of esthetics and (2) all types of forward- and backward-slanting profiles are esthetically less favorable [16]. In combination, these statements lead to the conclusion that AM Schwarz supposes the impact on facial attractiveness influenced by the sagittal position of the chin to be considerably more important than that of the midface, which has also been hypothesized by other authors [20, 29, 39, 40]. Our results only partially agree with these assumptions. Like previous studies, our results confirm that straight average faces were perceived particularly attractive and, thus, agree with the statements of AM Schwarz [3, 4, 15, 19, 41, 42]. However, not all types of straight profiles revealed equivalent ratings for attractiveness, as straight average faces were rated significantly more attractive than straight antefaces and these in turn significantly more attractive than straight retrofaces. Thus, our results contradict the first assumption of AM Schwarz. In addition, the very general statement that all types of slanting profiles are less attractive than straight profiles is only partially supported by our results: In our study, backward-slanting average faces and antefaces were rated comparably to the straight average face. In addition, the aggregated results revealed that the sagittal position of the midface was as decisive as the sagittal position of the chin for the assessment of attractiveness, contrary to AM Schwarz’s supposition. Our findings indicate that backward-slanting profiles were rated as attractive as straight profiles, while forward-slanting profiles were rated significantly less attractive. These data suggest that the direction of the sagittal discrepancy plays a greater role in the assessment of attractiveness.

In our study, retrofaces were rated the least attractive in terms of the sagittal position of the midface, and forward-slanting profiles in terms of the sagittal position of the chin, both characteristics commonly associated with class III malocclusions. This fact is consistent with the results of other investigations, showing that class III profiles were perceived significantly less attractive compared to straight profiles [4, 20, 32, 43–47]. Regarding profiles associated with class II malocclusion, evidence in the literature is less consistent. While some studies demonstrated that backward-slanting profiles were perceived as less attractive [4, 20, 32, 47–51], others did not confirm this correlation, similar to our results, or even observed higher levels of attractiveness in backward-slanting profiles [14, 45, 46, 52].

Previous research discussed these inconsistencies as a result of differences in the study cohorts due to cultural or ethnic background, gender, educational level, or professional background of the raters, which may have confounded the results [14, 43, 46, 52–60]. Therefore, we took those relevant contributing factors into account. Our results reveal that there were no clinically relevant differences in the perception of profiles between male and female subjects. When ranking the mean ratings given by females and males into ascending order, the slightly varying ratings given by both genders did not result in any differences. When summarizing existing literature, the impact of gender on the perceived attractiveness of profiles finally appears ambiguous, as there are as many studies that reported a significant effect [33, 61, 62] as those that did not [12, 15, 63]. Although existing literature does not allow for a definitive statement regarding the influence of education level, a higher level of education appears to result in more critical and differentiated assessments of attractiveness [15, 64, 65]. This is in line with the results of our study. On the one hand, we observed only slight mean differences between the different educational levels. On the other hand, profile types that were generally rated as particularly attractive or unattractive were rated more distinctly by subjects with a higher level of education, while subjects with a lower level of education tended to give more average mean ratings. Regarding the influence of cultural and ethnic background, there is a general consensus in the literature that this is a major factor in the perception of attractiveness [66–70]. Therefore, including only one ethnic group in studies aiming at assessing attractiveness to avoid inhomogeneity was recommended [44]. Consequently, the results of our study should only be applied in the context of Caucasians.

Some of the strengths of this study include the large study cohort of 1261 subjects, which is many times larger than the cohorts of previous studies in this context [4, 15, 19, 20, 24, 32, 36, 44]. By using the Basler face model to visualize facial profiles, we applied a method that combines a realistic representation of facial structures without showing excessive details, such as hair and skin texture. Various factors that might potentially confound the results were either controlled by including exclusively Caucasians or by performing a differentiated analysis according to gender and level of education. In addition, AM Schwarz’s hypotheses about the attractiveness of facial profiles have been validated for the first time, although this analysis method has been commonly used in Germany for several decades.

Nevertheless, the present study also includes some limitations. Despite our careful efforts to ensure that only the sagittal positions of the midface and chin were adjusted according to AM Schwarz’s instructions, these adjustments inevitably lead to changes in other parameters, such as the lip profile or the nasolabial angle, which are also known to affect the perception of profile attractiveness [6, 9, 19, 23, 37, 48–50, 71–73]. In addition, for all profile types that deviate from the straight average face, only one variant with moderate degree of sagittal displacement was investigated in this study. Our intention was to avoid overwhelming the subjects by presenting only a total of nine profiles. However, this approach limits our ability to evaluate how different severities of displacement further affect the results. For instance, backward-slanting profiles might also be perceived as significantly less attractive when a certain degree of displacement is exceeded. Future research should specifically address this aspect.

Facial appearance significantly influences various aspects of life, including self-esteem, social acceptance, professional opportunities and overall quality of life [5, 20, 38, 55, 63, 74–77, 77–80]. Therefore, it is not surprising that a primary motivation for many patients seeking orthodontic therapy is the wish to improve their facial esthetic appearance [4, 53, 55, 58, 71, 81–85]. The profile line (silhouette) is a major factor in the perceived attractiveness of a person [63, 73] and orthodontic therapies can significantly modify the facial profile through various effects, highlighting the responsibility of proper orthodontic planning [4, 5, 7, 8, 10, 15, 23, 66, 72]. This study highlights the impact of the sagittal positions of the midface and chin on facial attractiveness, contributing to a better understanding of the esthetic outcomes of orthodontic interventions.

## Conclusion

The findings of this study underscore the significant role of facial profile and chin position in shaping perceptions of attractiveness. Specifically, straight and backward-slanting profiles were rated as the most attractive, while forward-slanting profiles were deemed the least attractive. Additionally, average faces consistently received higher attractiveness ratings compared to both antefaces and retrofaces. Since orthodontic therapy influences the sagittal relationship of the jaws, it is crucial for orthodontists to be aware of these differences in perceived attractiveness, as it may influence treatment planning and improve patient outcome. However, given that the sample was drawn from a German population, the generalizability of these results to other ethnic and cultural groups may be limited. Given the influence of cultural and societal factors on the perception of attractiveness, further research across diverse populations is needed to expand our understanding of these factors on a global scale.

## Data Availability

This manuscript has no associated data.
